# *Trichomonas tenax*: A Neglected Protozoan Infection in the Oral Cavities of Humans and Dogs—A Scoping Review

**DOI:** 10.3390/tropicalmed8010060

**Published:** 2023-01-12

**Authors:** Maurice A. Matthew, Nawu Yang, Jennifer Ketzis, Samson Mukaratirwa, Chaoqun Yao

**Affiliations:** Department of Biomedical Sciences, One Health Centre for Zoonosis and Tropical Veterinary Diseases, Ross University School of Veterinary Medicine, Basseterre P.O. Box 334, Saint Kitts and Nevis

**Keywords:** *Trichomonas tenax*, periodontal disease, pathogenicity, control and prevention

## Abstract

*Trichomonas tenax* is a flagellated protozoan parasite found in the oral cavities of humans and animals and has been associated with periodontal disease, the most prevalent inflammatory disease affecting them all. Studies have shown that *T. tenax* can cause damage to mammalian cells and secretes virulent proteins, such as cysteine. It is presently considered zoonotic. Despite the few studies that have been done, the pathogenicity of this oral protozoan is still not fully understood. A database search was performed in July 2022 using PubMed and Google Scholar to retrieve data eligible for this study. PRISMA-ScR guidelines were followed to conduct this scoping review. A total of 321 articles were found with 87 included in this review after applying the exclusion criteria. Due to its increasing prevalence worldwide in both humans and dogs, detecting and elucidating the pathogenicity of this parasite is paramount for effective global control and prevention of periodontal disease. However, there is a paucity in the literature on this neglected zoonotic trichomonad, which is in large contrast to the closely related human pathogen *T. vaginalis*. Here, we comprehensively review the history, morphology and reproduction, host, prevalence, diagnosis, pathogenicity, control, and prevention of *T. tenax*. Hopefully, this article will call attention to both medical and veterinary professionals as well as epidemiologists on this most neglected and zoonotic protozoan. More epidemiological and clinical studies need to be conducted on *T. tenax* to gain a better understanding of its pathogenicity, to increase the chances of developing effective drugs to aid in the control of this oral parasite, and reduce the spread of periodontal disease worldwide.

## 1. Introduction

*Trichomonas tenax* is an anaerobic flagellated protozoan of an ancient eukaryotic lineage without mitochondria that lives in low-oxygen environments [1,2]. In contrast to its close relative, *Trichomonas vaginalis* found in the human urogenital tract, *T. tenax* is mostly found to inhabit the oral cavities of humans and animals, which may lead to periodontal disease [3]. The protozoan has also been found in other parts of the body, such as the lungs and bronchi, submaxillary glands, tonsils, and lymph nodes [4,5]. Despite its importance, relative to both human and animal health, very little attention has been paid to *T. tenax*. Therefore, this scoping review aims not only to identify future research gaps but also to call attention to both medical and veterinary professionals on the epidemiology, detection, and risk associated with contracting this greatly neglected oral protozoan.

## 2. Materials and Methods

### 2.1. Data Search Strategy

This scoping review was executed to assess the following research questions: (1) Is *T. tenax* more prevalent in humans and dogs with periodontitis compared to those that are healthy? (2) Is there an association between *T. tenax* and periodontal disease? (3) What techniques are presently available to detect *T. tenax*? (4) Does *T. tenax* exosomes contains virulent proteins and RNA, such as its close relative *T. vaginalis*? (5) Are there any treatments or measures available for the control and prevention of *T. tenax*? This scoping review was conducted by adapting the guidelines of the preferred reporting items for systematic reviews and meta-analyses extension for scoping reviews (PRISMA-ScR) [6]. A literature search was conducted on 29 July 2022 with an open date using PubMed and Google Scholar databases for published articles. The databases were searched for the keywords *Trichomonas tenax* and *Trichomonas elongata* (Table 1). The results were exported in .csv format and then opened and organized in Microsoft Excel.

### 2.2. Study Inclusion and Exclusion Criteria

**Figure 1:**
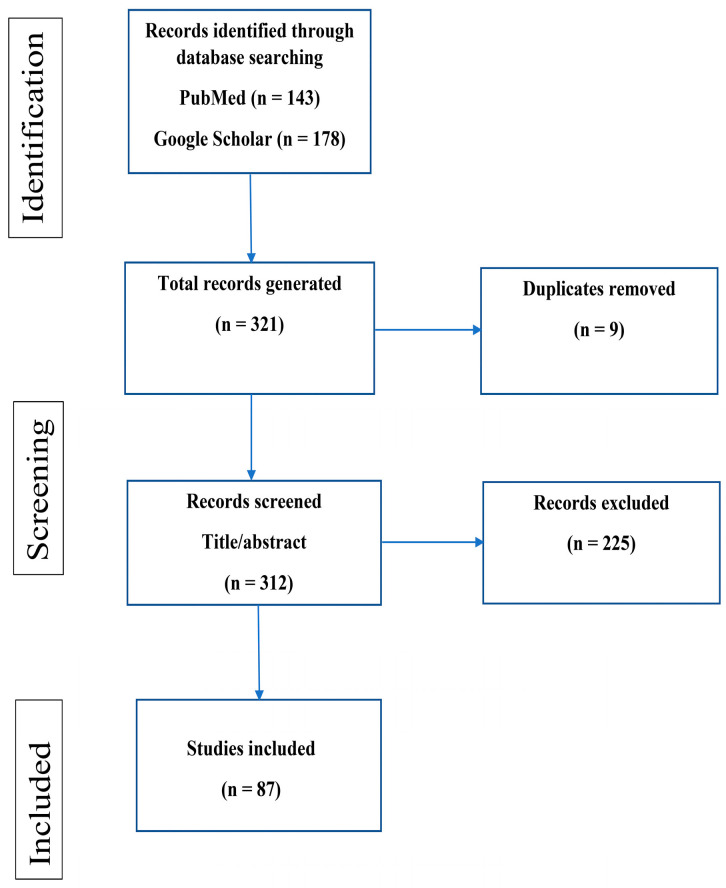
Flow diagram of scoping review selection process.

The inclusion criteria included all peer-reviewed published articles in English that included any information on the history, morphology, reproduction, host preference, the prevalence in humans and dogs, diagnosis, pathogenicity, and control and prevention of *T. tenax.* All articles were required to have an abstract and/or full text available to be included in this study. The search was not restricted to the publication date; therefore, all articles up to 29 July 2022 were included once inclusion criteria were met. Studies were excluded if no abstracts were available, if the article was not in English, if the article was not relevant based on the title and abstract, and if it was a duplicate (Figure 1).

### 2.3. Data Extraction

For data extraction, two reviewers, (MM) and (CY), independently read the title and abstract of each article retrieved from the search to determine if the article was eligible. Full-text screening was conducted by (MM), (CY), and (JK), and the data were extracted independently. Any differences or disagreements on eligibility were discussed and resolved by the team. The data extracted included the author, publication date, title, history, morphology, reproduction, host preference, the prevalence in humans and dogs, diagnosis, pathogenicity, and control and prevention.

## 3. Results

A total of 321 articles were retrieved after searching 2 databases (PubMed = 143; Google scholar = 178). Three hundred and twelve articles were screened after duplicates were removed. After inclusion and exclusion criteria were applied, 87 articles were eligible for this scoping review (Figure 1).

### 3.1. History

The name *Trichomonas tenax* originated from the Greek words “trichos” (meaning tiny hair), “monas” (meaning simple creature), and the Latin word “tenere”, which means to keep or to stick to [7]. The trichomonad protozoan was originally discovered by Muller in 1773, in aqueous solutions of tartar derived from the oral cavities of human beings [8]. He named it *Cercaria tenax* [9]. Up until the 1940s, different names were used, such as *Trichomonas elongata*, originating from Steinberg in 1862 [10], and *Tetratrichomonas buccalis* [11]. Although *T. tenax* was first observed by Muller in 1773, it was in 1917 that the first accepted name was given to the oral flagellate, which at that time was called *Tetratrichomonas buccalis* [11]. This was the first recorded report on the oral trichomonad in the 1900s. According to Honigberg and Lee (1959), it was Dobell (1939) and Wenrich (1944) who assumed that *Trichomonas* was the only flagellate in the buccal cavity and that its name should be *tenax* based on priority (hence, the name *T. tenax*) [9].

### 3.2. Morphology and Reproduction

Trophozoites of *T. tenax* are either ellipsoidal or ovoid (pear-shaped) in shape and 5–16 × 2–15 µm in size. Each has five flagella—four free anterior flagella and one that extends posteriorly. An undulating membrane extends two-thirds of the body length and its accompanying costa typically lies next to the posterior flagellum [12,13,14]. The four anterior flagella arise from a three-lobed blepharoplast, which gives rise to a chromatic basal rod and an axostyle. The nucleus is situated near the anterior end of the body and is generally ellipsoidal or ovoidal with an average size of 2.5 × 1.7 µm. It contains a single nucleolus, surrounded by clear halos [15,16]. The anterior end of the capitulum runs continuously with the pelta and there is no cell mouth present [9]. In addition to trophozoites, *T. tenax* has other forms. These forms include a round form that is usually larger than the trophozoite, an amoeboid cell form found swimming freely in axenic medium, and a pseudocyst form, which is found when the trichomonads are under stressful conditions [17]. These forms can range in size from approximately 5 to 16 µm in length and 5–6 µm wide [7,14].

In the past, these different forms of the trichomonad were considered different life-cycle stages. However, more recently, it has been shown that these different forms are due to different environmental conditions of the trichomonad’s habitat [17,18].

*Trichomonas tenax* can be readily differentiated from other species of the Trichomonadidae family, except for two, *T. vaginalis* and *T. gallinae*. Honigberg and Lee (1959) stated that from a morphological standpoint, *T. vaginalis* is on average larger than the other two trichomonads and the Paracostal granules are larger and more numerous compared to the other two trichomonads. Paracostal granules are mostly absent or small in *T. tenax* whereas in *T. gallinae* the granules are found in the region of the axostyle [9]. Furthermore, these trichomonads exhibit host and site predilection, with the *T. vaginalis* site of predilection being in the urogenital tract of humans, *T. tenax* in the oral cavities of humans and animals, and *T. gallinae* in the upper digestive tracts of avian hosts [19].

*Trichomonas tenax* reproduces by asexual reproduction, followed by a process of mitosis involving six morphologically distinct chromosomes [20]. Honigberg and Lee (1959) reported that during division, the parental flagella are divided equally among the daughter mastigonts with full flagellar completion taking place right before cytokinesis. Additionally, each daughter cell receives one of the parabasal, one daughter cell retains the old undulating membrane and costa while the other develops new organelles. By the end of the division, the old axostyle and pelta are destroyed and a new supporting organelle develops in both daughter cells [9].

### 3.3. Host

*Trichomonas tenax* has been cited and recorded in different animal hosts over the years. It was first reported in the oral cavities of humans by Muller in 1773 [8]. This was confirmed by other studies that also found *T. tenax* in the oral cavities of humans [21,22,23]. It has also been observed in samples taken from the vaginas of monkeys [24]. Several studies reported that *T. tenax* can cause urogenital invasions in humans in addition to its close relative *T. vaginalis* [25,26,27]. Dybicz et al. (2018) detected *T. tenax* in the oral samples of small companion animals, such as dogs and cats, as well as large animals, such as horses [28]. Other studies have reported on the occurrence of *T. tenax* in the oral cavities of dogs and cats [3,29]. Recently, *T. tenax* was detected in the cloaca of birds [30,31,32,33,34,35]. With *T. tenax* being found in so many different hosts, it is important to determine whether *T. tenax* is a human parasite, which is the predominant current view, or if it is a parasite of animal origin with zoonotic potential. This question on host preference and specificity requires further investigation.

### 3.4. Culture

Culturing *T. tenax* is important for studying the biochemistry, physiology, and metabolism of parasites, and to determine the nutrient requirements, morphological structure, pathophysiology, life cycle, and host–parasite relationship [13]. Before 1917, *T. tenax* was of little or no interest to clinicians and biochemists since it could not be maintained in an axenic culture unlike its close relatives, *T. vaginalis* and *T. gallinae* [9]. Although there were reports of *T. tenax* being cultured as early as 1915, these reports were not credible [36]. In 1917, Ohira and Noguchi continued culture work on *T. tenax*, but this work was also brief with no final conclusions as to whether the culture was successful or not [37]. Culture studies on *T. tenax* have increased over the years as they have (simultaneously) attracted the attention of researchers. Other culture work was followed by Hinshaw and Hogue in 1926, giving a better understanding of environmental conditions and nutrient requirements necessary to culture the oral trichomonad [38,39]. Honigberg et al. (1959) tried without success to culture *T. tenax* axenically [9]. Success was finally reached in 1962 when L. S. Diamond was able to induce axenic growth in a nutrient broth supplemented with serum and a cell-free extract of the chick embryo, which is now referred to as Diamond’s medium [40]. Currently, Diamond’s medium is still used by researchers worldwide to culture *T. tenax*. Although Diamond and Bargit (1962) were successful in culturing *T. tenax,* other studies continued as the oral trichomonad gained interest. Work by Wantland et al. (1963) and Asai et al. (1986) was somewhat unsuccessful but still contributed to the overall understanding of the oral flagellate [41,42]. Wantland et al. (1963) used egg yolk, a non-synthetic serum, to culture *T. tenax* and *Entamoeba gingivalis*, which resulted in the near-axenic and mono-axenic culture of *T. tenax* and *E. gingivalis*. Asai et al. (1986) used RPMI 1640 and Eagle’s minimum essential medium (MEM) to culture *T. tenax* but cultures failed to support the growth of *T. tenax* without bacterial growth. Additionally, researchers have shown that *T. tenax* isolated from humans can be successfully cultured if incubated at a temperature between 31 and 37 °C with a pH between 7.0 and 7.5 for 72 h [41,42,43]. All of these contributions to the culture of *T. tenax* were mainly from human strains with only one being successful, i.e., culturing *T. tenax* from dogs [3].

### 3.5. Molecular Diagnosis

Currently, microscopy and molecular methods are used in the diagnosis of *T. tenax* infection in humans and animals. Trichomonads of ellipsoidal or ovoid (pear-shape) shapes and 5–16 × 2–15 µm in size, as revealed by the microscopy along with the predilection site of the mouth, either directly or after being cultured, can be used as criteria for diagnosing *T. tenax* infection [19] (Table 2). However, molecular methods such as PCR are preferred for confirmation since they are considered more sensitive and specific than the conventional techniques of culture and microscopy [13,44]. PCR was first used to detect *T. tenax* in human oral samples in 1997 [1], where primers were designed for the 18S rRNA gene of *T. tenax*, which was aligned with *T. vaginalis* and *T. foetus* (Table 3). Kikuta et al. (1997) concluded that the method was specific to *T. tenax* and had a limit of detection of 100fg DNA or as low as 5 cells [1]. In comparison with the gold standard microscopy in the detection of *T. tenax* (in ten healthy individuals and nine patients of periodontitis or gingivitis), the PCR only detected *T. tenax* in five patients, whereas microscopy was negative for all patients and healthy individuals [1]. The data clearly show that PCR surpasses microscopy in sensitivity and yet maintains specificity. However, it was not until a case study by Szczepaniak (2016) that PCR was used to detect *T. tenax* in canine oral samples where primers for the ITS1-5.8S rRNA-ITS2 regions were used [45].

Over the last two decades, the loop-mediated isothermal amplification (LAMP), a nucleic acid amplification test, was developed to amplify the DNA of different pathogens for detection and diagnostic purposes [46]. This technique has been proven to be more sensitive than traditional techniques of culture and microscopy and PCR that are presently used [47,48,49]. It is rapid, does not require expensive equipment, and is ideal for future diagnoses, especially in developing countries [50,51]. To date, only one LAMP assay has been developed to detect *T. tenax* in human and/or canine oral samples, which can be directly used for clinical samples without prior DNA extraction. The test has a limit of detection of 10fg DNA or 1 cell [52]. A total of 8 out of 44 clinical canine samples were microscopically positive for *T. tenax* after culturing. They were also LAMP-positive when two cells were used in each reaction without prior DNA extraction [52]. Therefore, this LAMP assay has the great advantage of being used in point of care in both developed and developing countries. No other new molecular techniques have been reported in the literature to specifically detect *T. tenax* in humans and/or animals.

### 3.6. Prevalence

#### 3.6.1. Humans

The only reliable method available that could have identified or detected *T. tenax* in the 1900s and most of the 20th century was microscopy [38]. Hinshaw (1926) found that 90% of the prisoners with advanced pyorrhea (periodontitis) were infected with *T. tenax*. Several prevalence studies in humans have followed since that time (Table 4) [21,22,23,53,54,55,56]. In Iraq, the prevalence of *T. tenax* was 8.4% in 143 mouth disease patients and 4.1% in 271 controls [23]. Norberg (2014) reported the prevalence of both *E. gingivalis* and *T. tenax* in patients with oral infections in Brazil with the prevalence of *T. tenax* being 51% (51 out of 100 patients). The study by Norberg (2014) also showed that the flagellate infection decreased with age in the control group and increased with age in those who were ill. Additionally, *E. gingivalis* and *T. tenax* infections increased in individuals with tooth loss, indicating a positive correlation between tooth loss and both infections [21].

The difficulty in culturing and identifying the parasites made way for the development of molecular techniques, such as PCR and LAMP. Athari et al. (2007) reported on the prevalence of oral trichomoniasis in 160 patients with gingivitis and periodontitis using the PCR-amplifying 18S rRNA gene and microscopy. A total of 33 patients (20.6%) were PCR-positive whilst 28 (15.5%) were diagnosed microscopically. The study further found an association between the prevalence of *T. tenax* and the severity of periodontitis [57]. Mehr et al. (2015) investigated the prevalence of *T*. *tenax* in the periodontal lesions of patients who also had Down’s syndrome in Iran. Moreover, 52 patients were presented with periodontal disease and 52 with healthy gingiva; the prevalence was 14 (26.9%) and 5 (9.6%), respectively [58]. Dybicz et al. (2018) used PCR as a method of detection for *T. tenax* in patients with health issues, including diabetes, renal transplant, and rheumatoid arthritis. Healthy individuals were used as controls and the primers targeted the ITS1-5.8S rRNA-ITS2 region rather than the 18S rRNA, as mentioned earlier. The prevalence of *T. tenax* in the oral cavity of the control group was 10.2% (33 of 226), 14.1% (13 of 92) in diabetics, 12% (6 of 50) in renal transplant patients, and 14% (7 of 50) in rheumatoid arthritis patients. A higher prevalence of *T. tenax* was revealed in adults from all groups involved [4]. Two additional studies have been published, which used PCR to determine the prevalence of *T. tenax* in patients with periodontal disease. In one study, primers designed from the RNA polymerase II *rpbI* gene for *T. tenax* strain NIH4 [59] were used and the other study used primers designed from the β-tubulin gene of *T. vaginalis* [60]. Benabdelkader et al. (2019) included 50 patients in the study—20 with gingivitis and 30 with periodontitis. The overall prevalence of *T. tenax* was 56% (28/50). Interestingly, it was found to be more prevalent in patients with periodontitis than in those with gingivitis, i.e., 70% (21/30) and 35% (7/20), respectively [59]. In a later study using PCR by Bracamonte-Wolf et al. (2019), out of 106 periodontitis patients and 85 healthy controls, the prevalence was 34% (36/106) in periodontitis patients and 28% (30/85) in the control group [60]. Collectively, these data showed a strong correlation between *T. tenax* and periodontal disease in humans. Additionally, these data represent the evolution of techniques developed over the years to detect and assess *T. tenax* in humans.

The variability in the prevalence of *T. tenax* in humans is possibly due to the difference in the standards of oral hygiene in the different populations and due to the increased sensitivity of the detection methods. In populations with low oral hygiene and poor socioeconomic backgrounds, the prevalence of *T. tenax* is high compared to populations with average or good oral hygiene and socioeconomic backgrounds [14]. In a cross-sectional survey carried out in Iran, Azadbakht K et al. found that the odds ratio (OR) of individuals brushing their teeth was 0.43 (95% CI: 0.21–0.88) in comparison with those who did not brush. Further, the OR of people who resided in urban areas was 0.22 (95% CI: 0.1–0.47) compared with those in rural places [61].

#### 3.6.2. Domestic Dogs

*Trichomonas tenax* was first reported in canines in 1927 with 22 out of 23 canines testing positive [62]. It was not until there was a PCR method designed to detect *T. tenax* that studies on canines started to increase. This was probably due to the difficulty in culturing and detecting the flagellate by microscopy in a canine host. Alternatively, it may be due to a lack of interest in this protozoan in veterinary medicine (until recently). To date, only three recorded studies have been reported on the prevalence of *T. tenax* in canines using PCR (Table 5) [3,28,29]. The first study was reported by Patel et al. (2017) in the United Kingdom. A total of 92 samples were collected from canine dental plaque and screened for the presence of *T. tenax* and *Entamoeba* spp.; the prevalence of *T. tenax* was 56.2% (52/92) and *Entamoebae* spp. was 4.34% (4/92). Furthermore, the next-generation sequencing of healthy, gingivitis, early-stage periodontitis, and severe periodontitis samples showed the prevalence of *T. tenax* at 3.51%, 2.84%, 6.07%, and 35.0%, respectively. These findings were the first conclusive evidence of the presence of *T. tenax* in canine oral plaque [29]. Kellerová and Tachezy (2017) also investigated the occurrence of oral trichomonads in 111 domestic dogs and 122 cats using cell culture, PCR, and sequencing of the ITS1-5.8S rRNA-ITS2 regions. The prevalence percentages of *T. tenax* in dogs and cats were 8.1% and 4.1%, whilst for the different *Trichomonas* spp., they were 30.6% and 6.6%, respectively. The study also identified *T. brixi* as a new species. It concluded that dogs 3 years or older, as well as crossbred dogs, showed an increased prevalence of *T. tenax* [3]. Dybicz et al. (2018) used PCR to detect *T. tenax* in domesticated animals, such as horses, dogs, and cats. In the study, 142 dogs, 57 cats, and 102 horses were examined for the presence of *T. tenax*. The prevalence of *T. tenax* in canines was 4.92% (7 of 142). Additionally, 9 of 11 DNA sequences of trichomonad isolates showed 100% identity with *T. tenax* sequence obtained from the GenBank. The study concluded that oral trichomoniasis spreading between humans and domestic animals should be taken into consideration since the owners of three positive dogs also tested positive for *T. tenax* [4]. Studies on the prevalence of *T. tenax* in canines remain limited and more studies are needed worldwide.

### 3.7. Pathogenesis and Virulence Factors

Two systematic reviews on *T. tenax* have concluded that there is an association between *T. tenax* and periodontal disease [63,64]. The increase in the prevalence and association of *T. tenax* with periodontal disease from previous studies leads to questions concerning the pathogenicity of the oral flagellate. Extensive studies have been conducted on the pathogenicity of its close relative of the urogenital tract, *T. vaginalis*, and bacteria associated with periodontal disease [17,65]. However, the pathogenicity of *T. tenax* with regard to periodontal disease is far less documented [14]. To date, only seven studies have focused on the pathogenicity of *T. tenax,* and the proteins secreted by the oral flagellate that exhibit virulent characteristics. The first reported study on the pathogenicity of *T. tenax* was by Ribaux et al. (1979), who investigated the proteolytic activity of *T. tenax* in whole cells. Unfortunately, only the abstract could be obtained for this scoping review [66]. Ribaux et al. (1983) reported another study on the immunohistochemical location of fibronectin-like proteins on the cell surface of *T. tenax*. Two strains of *T. tenax* were used to establish an immunofluorescence staining procedure. The cells gave a positive fluorescence stain with anti-fibronectin anti-serum and the controls remained negative. They concluded that *T. tenax* produces fibronectin-like proteins that could be responsible for tissue adhesion [67]. These early works led other researchers to further investigate the proteolytic activity of *T. tenax*.

Bózner and Demeš (1991) carried out a study on the proteolytic activity in crude extracts and culture filtrates from *T. tenax* in SDS-polyacrylamide gels containing copolymerized gelatin. A total of seven distinct proteolytic bands were found, of which, three had molecular weights ranging from 35 to 56 kDa. These bands were SH-dependent, and their inhibitory sensitivities were characteristic of cysteine proteinases. The other four bands had molecular weights ranging from 76 to 270 kDa; these were SH-independent and were inhibited by a chelating agent, EDTA, suggesting they belong to the metalloproteinase family [65]. The authors continued studying the degradation of collagens I, III, IV, and V by extracellular proteinases of *T. tenax*. They concluded that the degradation of all four collagen types was temperature-dependent, with collagen IV being digested most effectively. They further stated that E-64 and the activation by reducing the agent dithiothreitol indicate that cysteine proteinases from *T. tenax* are responsible for the cleavage of collagen [68].

Another study that focused on the pathogenicity of *T. tenax*, carried out by Nagao et al. (2000), investigated the ability of *T. tenax* to lyse the red blood cells of sheep, horses, and humans. To achieve this, five fractions derived from intact cells, culture supernatant, cultural filtrate, cell debris, and lipid enriched fractions were used to assess the hemolytic activities under various conditions; only the culture supernatant was negative for hemolytic activity, the other four samples were positive for hemolytic activity. The authors concluded that the hemolytic activities were due to two types of hemolysins, one which is protein-like and the other lipid-like. The protein-like hemolysin was heat-labile and inhibited by various cysteine–proteinase inhibitors [69]. A decade later, El Sibaei et al. (2012) investigated the proteinase activities of seven isolates of *T. tenax* obtained from clinical patients in Egypt. The study also concluded that proteinase bands were observed, and these bands were intensified with a cysteine proteinase activator and disappeared completely in the presence of the cysteine proteinase inhibitor, further suggesting that the proteinases found were also cysteine proteinases [70]. These cysteine proteinases are the same virulent proteins that have been detected in *T. vaginalis* [71]. Recently, Ribeiro et al. (2015) performed a study where *T. tenax* fulfilled the requisites of a parasite, damaging different mammalian cells and behaving in a similar manner to *T. vaginalis* [17]. In short, limited work has been done on the pathogenicity of *T. tenax*, although there is growing evidence that it contributes to periodontal disease. Therefore, more studies are needed to better understand the pathophysiological processes of *T. tenax* infections in humans and other mammalian hosts with reference to periodontal disease.

Regarding the proteomics of parasites, exosome release has been gaining attention. Research has shown that many parasites excrete proteins enclosed in exosomes, which are considered potential virulent factors [72]. Exosomal studies performed on *T. vaginalis* have found that it secretes exosomes (such as those found in mammals) containing RNA and parasite-specific proteins [73]. Twu et al. (2013) illustrated that *T. vaginalis* exosomes deliver their contents to the host cell, modulating the cell’s immune response when fused. Additionally, the study was the first to show the potential role of exosomes in parasite-to-parasite communication [73]. Another study examined the major surface proteins (MSP) in the exosomes of the *Leishmania* spp. These proteins have been shown to digest extracellular matrix proteins. The study classified the MSP proteins released in *L. infantum* exosomes from promastigotes in avirulent procyclic (logarithmic), virulent stationary, and metacyclic stages, respectively, and found high levels of MSP in exosomes released from the stationary and metacyclic promastigotes than in the logarithmic promastigotes [74]. Work on exosomes is the new path that scientists are taking to gain a better idea of the pathogenicity of parasites. Therefore, it is important to investigate the pathogenicity of *T. tenax* by isolating exosomes, finding virulent factors by proteomic analysis, and elucidating exosome interactions with epithelial cells. This would be a significant contribution and addition to the pathogenicity of *T. tenax.*

### 3.8. Control and Prevention

Limited studies have been reported on the control and prevention of *T. tenax* despite its high prevalence among the human and canine populations. This could be because the pathogenesis of the oral protozoa remains unclear. However, studies have been documented on the treatment and prevention of its close relative *T. vaginalis* [73,75,76,77]. *Trichomonas tenax* can be transmitted between individuals by droplets from the mouth, kissing, or the use of contaminated dishes and drinking water [23]. Few studies have investigated treatment and prevention measures to control *T. tenax* infections. One study investigated the effects of non-surgical periodontal therapy (i.e., deep cleaning with scaling and root planning) on *T. tenax* and *E. gingivalis* in patients with chronic periodontitis. Rashidi Maybodi et al. (2016) reported that non-surgical treatment can reduce *T. tenax* and *E. gingivalis* in the oral environments of patients with chronic periodontitis [77]. Another study investigated the in vitro activities of selected mouth rinses on the reference strains of *T. tenax* and *E. gingivalis*. In this study, two standard strains of *T. tenax* (ATCC 30207) and *E. gingivalis* (ATCC 30927) were used, and metronidazole was used along with fourteen mouth rinses. The activities of the preparations were evaluated based on the ratio of dead to living cells after incubation at (37 °C) for 1, 10, and 30 min. The death of protozoa was categorized by the lack of movement and changes in the shape and characteristics of cell disintegration. The study concluded that all mouth rinses tested were effective on both protozoa [78]. There are no vaccines or drugs reported in the literature to effectively treat *T. tenax* infections in the oral cavity. However, metronidazole and tinidazole are the drugs approved by the U.S. FDA and EMA to treat vaginal trichomoniasis caused by *T. vaginalis* [72,73,75,79]. Since both protozoa are flagellates and closely related, there may be a possibility that metronidazole and tinidazole are effective at treating oral trichomoniasis caused by *T. tenax* as well [80]. Moreover, studies focusing on the treatment and prevention of *T. tenax* are needed since it is prevalent in both humans and animals that exhibit signs of periodontal disease [81]. It is worth noting that *T. tenax* has been found in the urogenital tracts of humans [25,26] and, hence, possibly contributes to human trichomoniasis (although how significant a contribution is to be determined).

## 4. Discussion

Periodontal disease is a public health concern for humans and dogs worldwide. For years, the disease has been associated with the oral flagellate *T. tenax*, although the cause and effect have not been confirmed, and much is unknown about the pathology of the parasite. *Trichomonas tenax* was first seen in the oral cavities of humans, and site and host predilections were assumed. However, it is now found in the lungs [82,83,84,85], lymph nodes [86], vaginal samples [27], and subhepatic abscesses [87]. When it comes to the host, *T. tenax* has been found in humans, dogs, birds, cats, horses, and monkeys. A parasite that has been found in so many different sites and hosts, with zoonotic potential, should not be neglected, especially since the transmission between these hosts is not yet understood. Studies have shown its parasitic and pathogenic capabilities when in contact with mammalian cells. Researchers are now questioning this protozoan zoonotic potential because of the wide range of hosts it has discovered [17,88]. Its prevalence is increasing in humans and dogs, with the latter needing more research. The prevalence of *T. tenax* ranges between 1 and 90% in humans and 8–96% in dogs (Table 4 and Table 5). This gradual increase in prevalence worldwide should not be taken lightly. The prevalence in humans is in accordance with results reported by Szczepaniak et al. 2016; however, the prevalence in dogs was higher than what was reported by Norberg et al. 2014, due to limited studies. Moreover, based on studies published so far, it is clear that there is a direct association between periodontal disease and *T. tenax* infection [89,90,91]. Presently, several methods can detect *T. tenax*, including culture, microscopy, PCR, and LAMP. Thus far, LAMP is the most sensitive and specific method used to detect *T. tenax*, with a limit of detection of one cell, followed by PCR, and then microscopy, which is considered a gold standard [1,52]. Virulent proteins have been extracted from *T. tenax* similar to the ones found in its close relative, *T. vaginalis* [65,66,67,68,92]. However, the pathogenicity is still largely unknown. The area of exosome studies has shown great potential in finding the pathogenicity of various eukaryotic cells. With this new area of proteomics and the recent publication of the *T. tenax* draft genome [93], we can possibly move closer to mapping the pathway of *T. tenax* and developing a possible drug therapy that can control and prevent the transmission of this oral flagellate.

## 5. Conclusions

Periodontal disease is a major public health concern worldwide with prevalence ranging from 1 to 90%; it was ranked the 11th most prevalent disease condition in the world in 2016 [94]. Presently, microscopy, PCR, and LAMP are the available techniques used to detect *T. tenax*, with the latter being the most sensitive. In this scoping review, gaps in the knowledge and areas of research on *T. tenax* are indicated. The evidence, however, suggests that not only does *T. tenax* play a role in periodontal disease but there is an association between both oral flagellate and periodontal disease patients. It is imperative that the mechanism in which *T. tenax* adheres (and causes damage) to gums be functionally elucidated. This knowledge could increase the ability to develop other effective drug therapies to control this parasite and potentially decrease the prevalence of periodontal disease.

**Table 3:** Molecular diagnosis of *Trichomonas tenax*.

Method	Target Gene	Primers	Expected Amplicon Size (bp)	Limit of Detection	References
PCR	18S rRNA	5′AGTTCCATCGATGCCATTC3′ 5′GCATCTAAGGACTTAGACG3′	862	100 fg or 5 cells	[1]
PCR	ITS1-5.8S rRNA-ITS2	5′GAGAAGTCGTAACAAGGTACG3′ 5′ATGCTTCAGTTCAGCGGGTCT3′	368	N/A	[4]
PCR	*rpb1* gene	5′GCTGTCATCTCTTGTGGGGCTG3′ 5′AAACTCATGGGAGCTGCTGGTTC3′	3048	N/A	[59]
PCR	*Beta-tubulin* gene	5′ATACTCTATCGTCCCATCTC3′ 5′GCCATCATGTTCTTGTTATCG3′	405	N/A	[60]
LAMP	ITS1-5.8S rRNA-ITS2	5′GTCATGATGTATGCAACTCCGG-TCCTCACACGATGAAGAACG3′ 5′GGTTAATCTTTGAATGCAAATTGCG-TGTACTGTTACACGCATGCTTCT3′ 5′ACATTATGCCACGTTCTTCATCG3′ 5′TGCGCTAAACTTGGCTTCGG3′ 5′AGCAATGGATGTCTTGGC3′ 5′GCAGACAACGTAAGTTTGT3′	N/A	10 fg or 1 cell	[52]

N/A—not available; LAMP—loop-mediated isothermal amplification.

**Table 4:** Prevalence of *Trichomonas tenax* in humans with or without periodontitis.

	Periodontitis Patients		Healthy Individuals			
Country or Region	No. Tested (Positive)	Prevalence (%)	No. Tested (Positive)	Prevalence (%)	Method * (M, PCR)	References
America						
Brazil	100 (51)	51	N/A	N/A	M	[21]
Chile	30 (21)	70	N/A	N/A	PCR	[60]
USA	350 (315)	90	N/A	N/A	M	[38]
Asia						
Indonesia	373 (19)	5.1	N/A	N/A	M	[95]
Iran	50 (3)	6	50 (0)	0%	M	[56]
Iran Iran	160 (34)	21	160 (3)	2	PCR	[57]
	52 (14)	19	52 (5)	3	PCR	[58]
Iraq Iraq	143 (12)	8	271 (11)	4	M	[23]
	383 (31)	8	N/A	N/A	M	[55]
Japan	9 (5)	56	N/A	N/A	PCR	[1]
Turkey Turkey	220 (2)	1	N/A	N/A	M	[22]
	107 (3)	3	N/A	N/A	M	[54]
Thailand	90(23)	25.6	94(3)	3.2	PCR	[96]
Europe						
Croatia	51 (18)	36	N/A	N/A	M	[53]
France	106 (37)	35	85 (16)	19	PCR	[59]
Poland	192 (26)	14	226 (33)	15	PCR	[4]

* M—microscopy; N/A—not available.

**Table 5:** Prevalence of *Trichomonas tenax* in a domestic dog with or without periodontitis.

	Periodontitis Patients		Healthy Individuals			
Country or Region	No. Tested (positive)	Prevalence (%)	No. Tested (positive)	Prevalence (%)	Method * (M, PCR)	References
Czechia	111 (9)	8	N/A	N/A	PCR	[3]
Poland	142 (7)	5	N/A	N/A	PCR	[28]
UK	92 (52)	56	20 (4)	20	PCR	[29]
USA	23 (22)	96	N/A	N/A	M	[62]

* M—microscopy; N/A—not available.

## Figures and Tables

**Table 1:** Results obtained from search strategies used in databases.

Database	Search Terms	Results
PubMed	*Trichomonas tenax* *Trichomonas elongata*	138 5
Google Scholar	*Trichomonas tenax* *Trichomonas elongata*	153 25

**Table 2:** Morphological characteristics of trichomonad protozoa found in human infections *^a^*.

	*Trichomonas vaginalis*	*Trichomonas tenax*	*Pentatrichomonas hominis*	*Tritrichomonas foetus*	*Tetratrichomonas gallinarum*	*Trichomonas gallinae*
Definitive host	Humans	Humans, dogs, etc.	Humans, cattle, etc.	Cattle	Bird	Bird
Predilection site	Urogenital tract	Mouth cavity	Intestine	Urogenital tract	Intestine	Intestine
Shape	Piriform	Oblong	Pear-shaped to round	Spindle to pear-shaped	Fusiform to round	Pear-shaped to round
Size (µm)	7–32 × 5–12	5–16 × 2–15	8–20 × 3–14	10–25 × 3–15	6–15	12.5–20
Anterior flagella	Four	Four	Five	Three	Four	Four
Recurrent flagellum	Extends about 2/3 of the body	Ending posterior to the middle of the body	Entire length of the cell and beyond	Entire length of the body and beyond	Entire length of the cell and beyond	Almost the entire length of the body
Free posterior flagellum	N	N	Y	Y	Y	N

***^a^*** Adapted from [19].

## Data Availability

Not applicable.
